# Disitamab vedotin: a novel antibody-drug conjugates for cancer therapy

**DOI:** 10.1080/10717544.2022.2069883

**Published:** 2022-05-04

**Authors:** Fan Shi, Yanli Liu, Xuexiao Zhou, Pei Shen, Ran Xue, Min Zhang

**Affiliations:** a State Key Laboratory of Military Stomatology, Department of General Dentistry and Emergency, School of Stomatology, Air Force Military Medical University, Xi’an, China; b School of Stomatology of Qingdao University, Qingdao, China; c Department of Pharmacy, The First Affiliated Hospital of Xi ’an Jiaotong University, Chang’an District Hospital, Xi ’an, China

**Keywords:** Antibody-drug conjugates, human epidermal growth factor receptor 2, disitamab vedotin, trastuzumab emtansine, trastuzumab deruxtecan

## Abstract

Human epidermal growth factor receptor 2 (HER2) regulates cell mitosis, proliferation, and apoptosis. Trastuzumab is a HER2-targeted monoclonal antibody (mAB), which can prolong the overall survival rate of patients with HER2 overexpression in later periods of gastric cancer and breast cancer. Although anti-HER2 monoclonal antibody has a curative effect, adjuvant chemotherapy is still necessary to upgrade the curative effect maximumly. Antibody-drug conjugate (ADC) is a kind of therapeutic drug that contains antigen-specific antibody and cytotoxic payload, which can improve the survival time of tumor patients. To date, there are several HER2-ADC products on the market, for which two anti-HER2 ADC (trastuzumab emtansine and trastuzumab deruxtecan) have been authorized by the FDA for distinct types of HER2-positive carcinoma in the breast. Disitamab vedotin (RC48) is a newly developed ADC drug targeting HER2 that is comprised of hertuzumab coupling monomethyl auristatin E (MMAE) via a cleavable linker. This paper aims to offer a general insight and summary of the mechanism of action and the currently completed and ongoing clinical studies of RC-48 in HER-2 positive solid tumors.

## 1. Introduction

Human epidermal growth factor receptor 2 (HER2) is often expressed in a variety of tumors tissues and is closely related to the development of carcinoma, while HER2 activates the downstream signaling pathways through heterodimer and tyrosine kinase autophosphorylation mediated signal transduction (De Santis et al., 2019; Akbari et al., 2020); and which gene magnification and protein overexpression play a crucial role in the cell proliferation, adhesion, aggressiveness apoptosis as well angiogenesis of numerous solid tumors (Kaur & Dasanu, 2011). In addition, HER-2 can also build heterodimers with the rest of the EGFR family and produce a marked effect in regulating tumor cell proliferation, differentiation, migration, and tumorigenesis (Rohlenova et al., 2016; Choi et al., 2020). Approximately 15–20% of gastric carcinoma/gastric and gastroesophageal junction carcinoma (GC/GEJC) and 12–23% of breast cancers (BC) overexpress HER2 (Study Group HER2 Monitor, 2011). HER2 is overexpressed and/or amplified in a wide variety of malignancies, and it has been considered to have a worse prognosis. Correspondingly, HER2-directed therapies have proven to be very effective and thus, have significantly improved the survival of patients with BC.

Trastuzumab, the firstly generated monoclonal antibody (mAB) that targets HER2 and which could enhance the overall survival of HER2 patients overexpressing advanced GC/GEJC (Aoki et al., 2021]. The mAB has an outstanding curative effect on cancer cells but its killing effect on carcinoma cells is limited. Therefore, the combination of anti-HER2 drugs and chemotherapy drugs can produce the highest antitumor effect. In addition, HER2 overexpressed cells have a high proliferation rate, causing a significant response to cytotoxic drug treatment. Besides, the cytotoxic drug has a strong effect on the destruction of tumor cells, and it will also significantly affect every dividing cell, containing those in the normal tissues, resulting in serious adverse reactions (Wolska-Washer & Robak, 2019). Given the integrated efficacy of HER2-inhibition and chemotherapeutic drugs, scientists used a linker to unite the two segments to obtain ADC. ADC includes bioactive cytotoxic drugs and monoclonal antibodies (mAb) that resist the target antigen, by chemical bonds (a linker) and which are devised for transmitting cytotoxic agents to tumor cells, monoclonal anti-body acts as a transporter, targeting cytotoxic drugs into specific cells (Zhu et al., 2021).

To date, there are several HER2-ADC products on the market, two anti-HER2 ADC for different indications have been authorized by the FDA for the therapy of HER2+ BC. Trastuzumab emtansine (T-DM1) was the first ADC that targeted HER2, which has been authorized by the FDA to treat HER2+ metastatic BC and to improve patient prognosis. However, the effect is unsatisfactory in the treatment of GC (Montemurro et al., 2020). As the newly launched drug in 2020, Trastuzumab deruxtecan (DS-8201a) is currently the most effective ADC drug that targeting HER2, which has obtained accelerated approval from FDA and conditional authorization from EMA for advanced or metastatic HER2+ BC patients who have received anti-HER2 drugs before, containing T-DM1 (Doi et al., 2017; Modi et al. 2020b). However, DS-8201 has not been approved for treating locally advanced and metastatic HER2+ GC/GEJC. Disitamab vedotin (RC48) is an innovative anti-HER2 ADC, including hertuzumab (a novel anti-HER2 mAB) coupling monomethyl auristatin E (MMAE) by a cleavable linker. But even more important, RC48 has been approved with the condition for the therapy of cancer patients with HER2+ (IHC2+/3+) locally advanced or metastatic GC/GEJC and UC who have been treated with systemic chemotherapy agents at least twice (Deeks, 2021).

## 2. The characteristics and therapeutic mechanism of ADC

**Figure 1.**
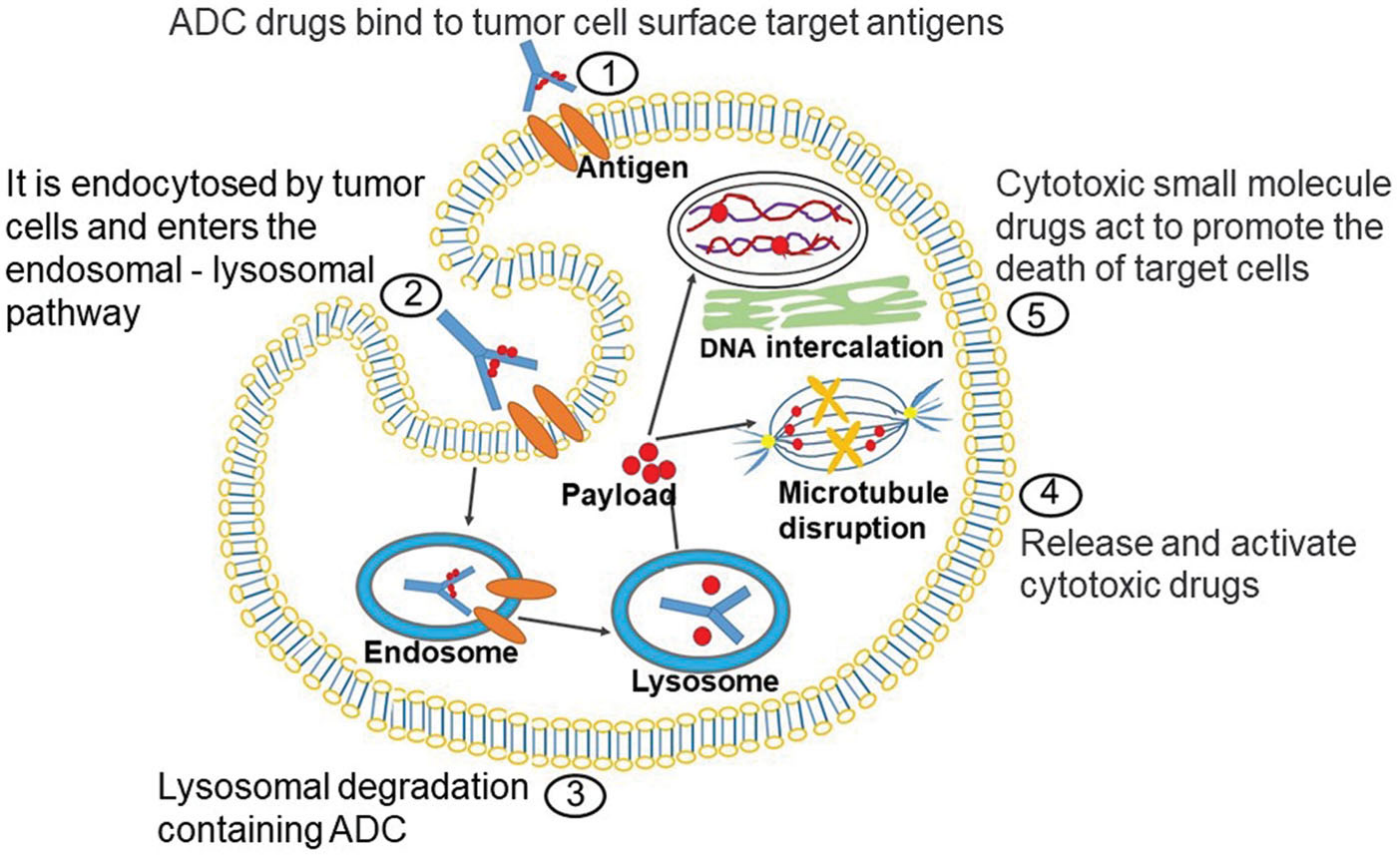
Action mechanism graphic of ADC. ADC consists of three structural sections: antibody, payload, and linker. The monoclonal antibody can be specifically distinguished by the antigen on the cell surface, and ADC gets into the target cell via an endocytosis process. The cellular proteases cleave the linker to release cytotoxic drugs that specifically kill the target cancer cells.

As the combinations of antibodies and highly potent cytotoxins, ADC can improve the therapeutic index and reduce the systemic toxicity related to cytotoxic payload (Amani et al., 2020). ADC usually consists of the following parts: antibody, cytotoxic payloads, and linkers. The design characteristics and anticancer mechanism of ADC drugs are as shown (Figure 1).

### 2.1. Antibody

Target spots refer to the tumor surface target antigen recognized by the antibody, and its selection determines the indication for the ADC drug, while the antibody specifically recognizes tumor surface target antigens and mediates the localization and endocytosis of ADC drugs in tumor cells.

### 2.2. Cytotoxic payloads

Cytotoxic payloads should have the following characteristics: firstly, which should be properly fat-soluble; secondly, the target should be located inside the cell; thirdly, cytotoxic payloads should be stable in the blood; last but not least, the cytotoxic payload molecules include the following characteristics: small dimensions, lack of immunogenicity, easy to dissolve in aqueous solution so that it is conducive to their combination. Currently, cytotoxic drug effector molecules contain microtubule inhibitors, DNA damaging agents, and DNA transcription inhibitors. Small molecule cytotoxic drugs with high toxicity and paralegal effects, after their release, binds to the corresponding action site, thus exerting a strong killing effect on tumor cells.

### 2.3. Linker

The linker is a component of the ADC that determines the drug delivery mechanism, pharmacokinetics, curative effect, and safety profile of the ADC. The most familiar connectives are the following two: cleavable linker and uncleanable linker. Cleavable Linker is sensitive to the intracellular environment, where it releases free effector molecules and antibodies through catabolism and dissociation in the cell. They are generally stable in the blood, but speedy lysate in low pH and protease-rich lysosomal environments and release effector molecules. In addition, if effector molecules can cross membranes, tumors can be eliminated through the potential bystander effect. An uncleanable linker is a novel developed linker with superior plasma stabilization than a cleavable linker. Owing to uncleanable linkers offer superior stability and safety than cleavable linkers, and these linkers depress off-target toxicity and offer a broader therapeutic scope. As is well known, the linker to realize the efficient release of cytotoxic drugs in tumor cells by precise release of toxic small molecules into cancer cells to kill tumor cells. At the same time, it will keep stable in the blood circulation and in tissues outside the target tissue to avoid the adverse reactions caused by the release of toxic small molecules. The coupling way directly determines the drug antibody ratio, the distribution of conjugation sites, and the stability of conjugation.

## 3. Disitamab vedotin

### 3.1. Chemistry of disitamab vedotin

**Figure 2.**
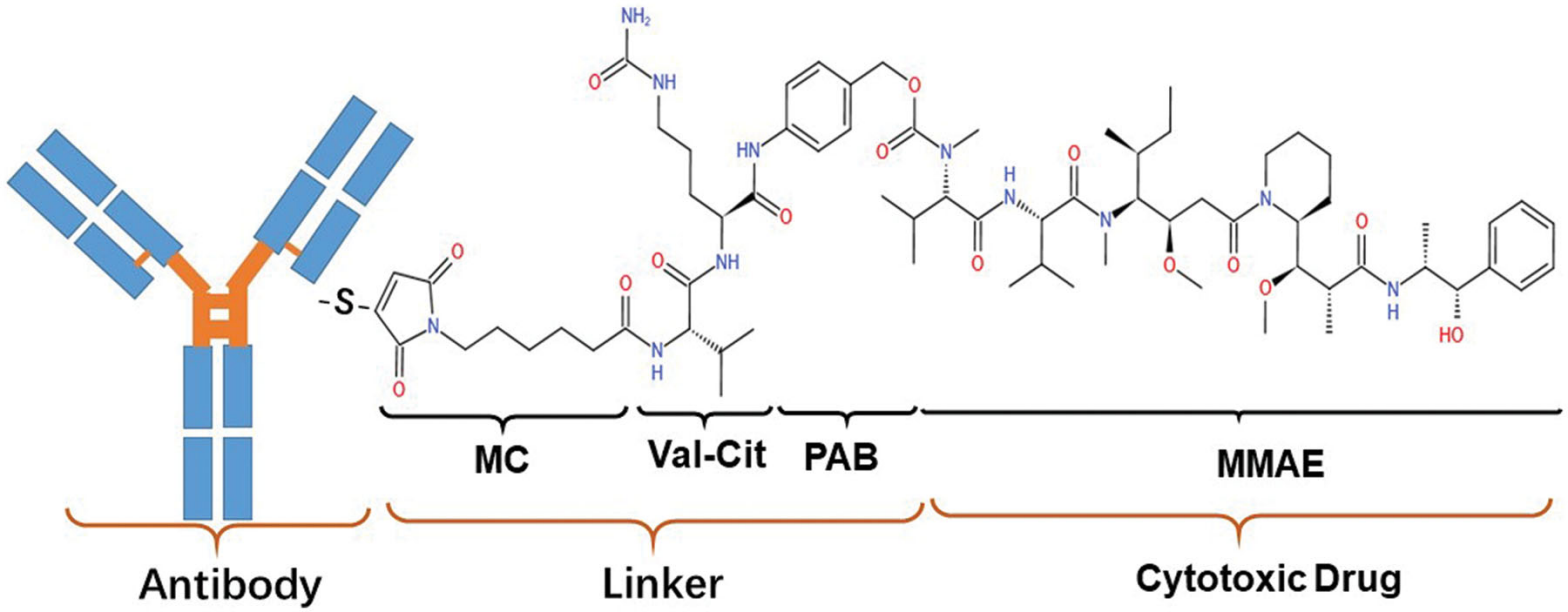
The molecular structure of RC48. These derivatives comprise antibodies (disitamab) and cytotoxic drugs (monomethyl auristatin E, MMAE) linked by valine-citrulline (VC).

RC48 is an innovative HER2-targeting conjugate, and this new ADC consists of MMAE, and HER2 antibodies via a valine-citrulline linker (Zhu et al., 2021). RC48, shown in Figure 2, is spanned into three components: antibody, payload, and linker. Antibody Disitamab——Disitamab targets different epitopes of the HER2 receptor and has a better molecular affinity for the HER2 target than trastuzumab. Linker——The valine-citrulline (VC) linker is stable, and can only be cleaved by cathepsins when RC48 are endocytosed into lysosomes, resulting in the release of payloads to kill target cancer cells (Bargh et al., 2019). payload——MMAE, a synthetic derivative of auristatin which has an anti-mitotic effect (Buckel et al., 2015).

### 3.2. Mechanism of action

**Figure 3.**
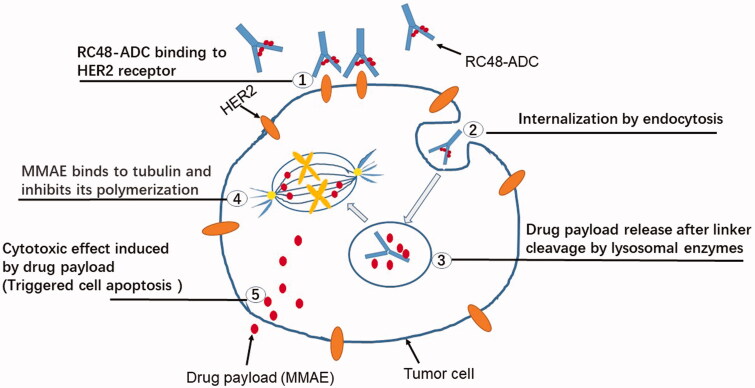
The anticancer mechanism of RC48. HER2 is highly expressed on tumor cells, and RC48 has a high affinity with HER2 to formulate the RC48-HER2 complex. Once the RC48-HER2 complex is internalized, MMAE is generated from Hertuzumab-VC-MMAE, and unites to β-tubulin, preventing cellular fission by suppressing microtubule assembly, leading to apoptosis and cell death.

The main detailed mechanism of action of RC-48 is the alternative delivery of anticancer agent MMAE to HER2-expressing cancer cells, anchoring HER2-protein on the surface of the tumor, RC48 precisely recognizes and binds to tumor cells as well which then kills tumors by penetrating their cell membranes. The following mechanisms are included (Figure 3). (1) Specifically binds tumor cells and mediates endocytosis: The HER-2 antibodies of RC-48 bind with high molecular affinity to the extracellular realm of HER2 on the surface of malignant cells, subsequently causing clathrin-based and caveolin-based endocytosis of RC48-ADC, which is further transported to lysosomes (Li et al., 2020). (2) Enzyme digestion and release of toxins: In the acidic environment of the lysosome, the activated lysosomal enzyme specifically digested the linker of RC-48 monoclonal antibody, releasing the covalently linked cytotoxic pentapeptide small molecule - tubulin depolymerizing agent MMAE. (3) Toxin-mediated apoptosis of tumor cells: The MMAE released inside the cell binds to microtubules or tubulin, damaging the microtubule structure inside the cell, further leading to mitotic cell cycle arrest and apoptosis. Moreover, RC-48 also interferes with the transcription, growth, and proliferation of tumor cells by inhibiting downstream signaling pathways activated by HER2.

### 3.3. Pharmacokinetics

The most common way for drugs to enter cells is by pinocytosis. Research by Xinan Sheng *et al.* (Li et al., 2020) shows that the amount of ADC entering the cells was decreased by inhibiting pinocytosis, and with inhibition of clathrin and caveolae, intracellular uptake of RC48 decreased significantly. In addition, besides pinocytosis, clathrin-mediated and caveolin-mediated endocytosis were additional uptake pathways for naked antibodies and RC48, as it has been observed for the uptake of trastuzumab (Buckel et al., 2015). Serum concentrations of RC48 fast-rising after intravenous infusion, and Jing Jiang *et al.* (Jiang et al., 2016] examined the release level of free MMAE in both serum and tumor, the result indicated that the concentration of total antibodies (T_Abs_) and released MMAE changed in serum and cancer through time, and in terms of the peak-times (T_max_) for T_Abs_ and MMAE in the neoplasm were slower than that in serum at different doses in a cancer model. Yingying Xu *et al.* (Xu et al., 2021) have conducted a dose-escalation and assessed the PK of RC48 in patients with HER2-positive GC, collected the value of PK of TA, binding antibody (BA), as well free MMAE (FM) in serious blood samples, and directly achieved the PK parameters, according to the PK profile data of TA and BA, both exposures and half-lives proportional added in a dose-dependent manner; more importantly, the PK profiles of TA and BA were homologous, which may demonstrate that the RC48 would be steady in serum; while showed that RC48 increased the release of MMAE at the cancer tissue (Jiang et al., 2016). The significantly lower C_max_ and AUC 0 − inf of FM in contrast to those of conjugate MMAE indicated that most of MMAE were Con-max 0 − inf of conjugate MMAE, which manifested that the majority of MMAE was coupled with antibody (Xu et al., 2021). And in cynomolgus monkeys, the plasma concentration of RC48 reduced exponentially after a single-shot intravenous dose (Jiang et al., 2016). It was speculated that free antibodies (FA) are gently released from RC48 after the process of cellular utilize, lysosomal internalization, and degradation *in vivo*, while the concentration of TA and BA exhibited a similar decline characteristic, therefore TA and BA possessed approximately the same half-lives; in short, the serum concentration of RC48 gradually decreased with time, whereas RC48 concentration in the tumor tissues remained more stable (Xu et al., 2021). The analysis of population PK demonstrated that the distribution volume of MMAE in peripheral compartments was significantly higher than that in the central ventricle(29.0 and 59.3 L, respectively) (Deeks, 2021). At different single doses, the half-lives of MMAE-bound antibodies showed a dose-dependent manner while the half-lives of free MMAE decreased with increasing doses (Deeks, 2021).

When it comes to drug antibody ratio (DAR), the existing study analyzed hertuzumab coupled with different amounts of drugs (including two, four, six, and eight drugs) per antibody (D2, D4, D6, and D8), results demonstrated that compared with D2 Hertuzumab ADCs, D4 had the equivalent potency and effect with approximately half the IC50, and there was almost no enhance in therapeutic effect when the quantity of conjugated linker-drugs was larger than D4 (Jiang et al., 2016). The ADC effectiveness mainly relied on drug loading profiles *in vitro* settings. However, in vivo antitumor activities of DAR-4 RC48 were exhibited to resemble DAR-8 RC48 at equal mAb quantity (Yaghoubi et al., 2021). Therefore, RC-48 with DAR 4 is optimal, and the half time (t_1/2_) and definite results of the PK characteristic of RC48 in rats were DAR-dependent (Jiang et al., 2016).

### 3.4. Pharmacodynamics

The anti-tumor effect of RC48 occurs via inhibition of the HER2-receptor signal pathway, MMAE-induced microtubule inhibition, apoptosis, and in vitro research data suggest RC48 may also have the characteristics of antibody-relied and cell-mediated cytotoxicity; in addition, alike all protein pharmaceutical drugs, RC48 has the potential for immunogenicity (Deeks, 2021). Previous research has pointed out that in the low-dose group, the Cmax and exposure of MMAE in the tumor tissues were 33- and 260-fold higher than that in the serum, respectively; in the high-dose group, they were higher by 16- and 187-fold, respectively. The difference was that in the same dose group, peak concentrations of MMAE in tumor tissues and tumor exposure to MMAE were higher than those in serum. These data suggested that MMAE was better released in the tumor tissues and showed targeted cytotoxicity (Li et al., 2020; Yaghoubi et al., 2021). MMAE was concentrated in tumor tissues, perhaps because after reaching the target site, RC48 was degraded into a toxin, which was then released, and as more antibodies entered the target site and MMAE continued to be released, MMAE accumulated in the tumor tissues, thereby resulting in high, tumor-specific cytotoxicity. The difference between the MMAE serum AUC and tumor AUC after the administration of the drug in the form of ADC showed that the serum exposure of MMAE can be reduced while greatly increasing tumor exposure, while the PK result of MMAE showed that RC48 had a high efficiency for tumors and low systemic toxicity. Furthermore, it demonstrated that the cancer suppression dose of hertuzumab-VC-MMAE was significantly better than T-DM1, and the former was almost three times that of the latter; research on drug concentrations of total RC48-ADC and released MMAE in cancer tissues and serum showed that the RC48-ADC was targeting HER2 and released MMAE at the cancer site to maintain a steady concentration level for one week in HER2+ ovarian cancer models (Jiang et al., 2016).

Tumor cells within the same carcinoma can exhibit different phenotypes and morphological characteristics, such as gene expression level, metabolism, cellular morphology, and motility as well metastatic potential (Swanton, 2012; O'Connor et al., 2015). The binding affinity for dissolved HER2 antigen showed equal avidity for hertuzumab coupling and noncoupling mAbs. Besides, the PK and PD of the conjugates exhibited a relation between effect and drug concentration (Jiang et al., 2016). Furthermore, in terms of heterogeneous cancer, directly killing the antigen-positive cells, some ADC can attack the adjacent antigen-negative cancer cells, such a prominent phenomenon is called the bystander killing effect, which is mainly relied on membrane penetration of the payload (Ogitani et al., 2016). The MMAE released by RC48 showed that membrane penetration could induce remarkable bystander effects, which can enhance efficacy on solid tumors (Moody et al., 2015; Amani et al., 2020). Namely, the increased expression level of HER2 promotes the uptake of conjugated MMAE and enhances its release in cells; FM can further attack carcinoma cells through the bystander effect (Li et al., 2020).

## 4. Therapeutic efficacy of RC-48 ADC in the clinical study

### 4.1. Gastric carcinoma/gastroesophageal junction adenocarcinomas carcinoma

The demonstrated HER2+ rate in gastric/gastroesophageal junction cancer ranges from 4.4% to 53.4%, and 22% of gastric cancer patients have high HER-2 expression, and about 24% of gastric cancer patients have low HER-2 expression. As is well known that HER-2 status determines GC/GEJC that benefit from targeted therapy, HER2+ carcinoma is supposed to have invasive biologic behavior (Subasinghe et al., 2019).

#### 4.1.1. Phase I studies of RC48 in HER2+ GC/GEJC

In phase I clinical study of RC48 in the therapy of solid cancer, the subgroup analysis of GC demonstrated that RC48 has outstanding anti-cancer ability on HER2+ GC, and its ORR and DCR reached 21.3% and 46.8%, respectively (Xu et al., 2021). Furthermore, among the eligible patients included in this study, those who had not received prior treatment with HER2-targeted therapy and those who had previously accepted HER2-targeted therapy had ORR of 25.9% and 15.0%, respectively, and DCR of 48.1% and 45.0%, respectively (Xu et al., 2021).

#### 4.1.2. Phase-II studies of RC48 in HER2+ GC/GEJC

A total of 127 eligible GC/GEJC patients were recruited for an open-label, single-arm, multicenter Phase-II clinical study with clinical characteristics: HER2 status is IHC2+/3+, regardless of fluorescent in situ hybridization amplification (FISH) status, disease progression after conferring 2 prior therapies, of which including 60 patients who had endowed at least 3 lines previous therapies, patients agreed to receive accepted RC48 2.5 mg/kg every 2 weeks until disease progression or intolerable toxic side effects led to discontinuation or death, and the results indicated that ORR was 24.4%, accompanying median duration of response (DOR) was 4.7 months, meanwhile median progression-free survival (m-PFS) and overall survival (OS) were 4.1 months and 7.9 months, respectively (Peng et al., 2021).

#### 4.1.3. Rc48 in HER2 low expressed GC/GEJC

Previous studies have shown that HER2 low expression (IHC2+/FISH−) exceeds 40–60% of GC patients, but no HER2-targeted ADC drugs are authorized up to now (Moelans et al., 2011). The results of the RC48 phase-I study of treatment of HER2 low expressed GC indicated that the antitumor response of HER2 IHC2+/FISH- patients resembled that of IHC2+/FISH + and IHC3+ patients, with 72.7%, 60.0% and 52.6% of patients achieving significant cancer shrinkage, separately (Xu et al., 2021). Therefore, as far as this study is concerned, it can be concluded that RC48 has a certain anti-cancer effect in patients with HER2 low expression GC.

### 4.2. Breast carcinoma

The majority of HER2-targeted therapies approved or in clinical development target BC patients with high levels of HER2 expression, while the more common type of BC is the low HER2 expression type, which accounts for about 50% of newly diagnosed cases (Pondé et al., 2019; Mahtani et al., 2020). Meghdad Abdollahpour‐Alitappeh *et.al* (Abdollahpour-Alitappeh et al., 2019) combined trastuzumab and microtubule‐disrupting agent MMAE to form a new type of ADC through a valine‐citrulline peptide linker for the treatment of BC. The results of their experiments showed that the new ADC (trastuzumab‐MC‐Val‐Cit‐PABC‐MMAE) and parental mAb had similar affinity on HER2-positive cells, while the ADC can significantly induce the death of HER2+ tumor cells, but no response to HER2‐ cells *in vitro*. It shows that MMAE‐conjugated trastuzumab can not only remarkably enhance the cytotoxicity of trastuzumab, but also display the advantages of high affinity, specificity, and anti-tumor activity in HER2+ tumor cells.

Calculation of curative effect of RC48 in patients with locally advanced metastatic BC, a dose-escalation phase I clinical study (NCT02881138) (Cullinane et al., 2020) has been completed, the dose range of RC48 in HER2+ (IHC 3+ or 2+/FISH+) BC patients is 0.5-2.5 mg/kg, and an open-label, parallel Phase Ib clinical trial (NCT03052634) (Modi et al., 2020a) in HER2+ BC patients who used 1.5-2.5 mg/kg RC48 every 2 weeks, while HER2-low expressing BC (IHC2+/FISH − or IHC 1+) patients used 2.5 mg/kg RC48 every 2 weeks, the results of the above studies were analyzed together, and the results demonstrated 31.4% of patients with HER2+ BC accomplished an ORR is 31.4%, while the median PFS was 5.8 months. In addition, the company is conducting phase Ib and Phase II/III registered clinical trials of RC48 in metastatic breast cancer in China.

In 2021, ASCO announced the latest data of 70 patients with HER2+ BC and 48 patients with HER2-low expression BC, the trial results show that RC48 can achieve good efficacy in both HER2+ and low-expression BC patients, and there are no new safety problems, while the 2.0 mg/Kg dose group had the best benefit-to-risk ratio, with m-PFS of 6.3 months.

### 4.3. Urothelial cancer

Following BC and GC, the third tumor type that HER2+ is UC, while the expression level of HER2 in patients was 48% with overexpression and approximately 20% with low expression (Fleischmann et al., 2011; Yorozu et al., 2020).

#### 4.3.1. Phase-I studies of RC48 in HER2+ UC

In a phase-I clinical trial, four eligible patients were recruited for phase I clinical study and was treated with RC48, 2 patients had partial remission and 2 with stable disease, and their corresponding ORR and DCR were50% and 100%, respectively. The results preliminarily show that RC48 has obvious anticancer advantages in UC (Xu et al., 2021).

#### 4.3.2. Phase-II studies of RC48 in HER2+ UC

In phase-II, an open-label, multicenter, single-arm study (NCT03507166) of RC48 in UC, 43 eligible patients were recruited with clinical characteristics: locally advanced metastatic UC, who had previously received at least first-line systemic chemotherapy and failed, every 2 weeks received 2.0 mg/kg of RC48, and the results were analyzed at a median follow-up time of 20.3 months, the ORR and the median DOR reached 51.2% and 6.9 months, respectively, while the m-PFS was 6.9 months and OS was 13.9 months (Sheng et al., 2021). In a similar clinical trial (NCT03809013), RC48 was administered to patients with HER2-overexpressing locally advanced or metastatic UC who had failed prior therapy with platinum, gemcitabine, and taxane treatment, RC48 also had significant antitumor potential, and its data showed and the evaluated ORR (primary endpoint) was 46.9%, the m-DOR was 8.3 months, while the m-PFS and m-OS were 4.3 months and 14.8 months, separately (Sharma et al., 2017). Given these data, the US FDA granted RC48 breakthrough therapy status for the treatment of HER2+ UC.

#### 4.3.3. Rc48 in HER2-negative UC

An open-label, single-center, single-arm Phase-II clinical study (NCT04073602) enrolling a total of 8 eligible patients with clinical characteristics: locally advanced metastatic UC, low HER2 expression (IHC of 0 or 1+), and prior receipt of at least one systemic therapy, had given consent to receive 2.0 mg/kg of RC48, at the data cutoff, the ORR of the statistical analysis was 25% and the DCR was 75% (Sharma et al., 2020). In addition, in a phase Ib/II clinical study (NCT04264936) of RC48 joint toripalimab in patients with locally advanced metastatic UC, after patients received 1.5 or 2.0 mg/kg RC48 plus 3.0 mg/kg toripalimab in the dose-increase stage; during the dose-enlarged stage, patients received 2.0 mg/kg of RC48 plus 3.0 mg/kg toripalimab every 2 weeks, at the data cutoff time, the ORR is 80% and the DCR is 90% after the data are staged together (Li et al., 2019). The above study results indicated that RC48 combined with terriprizumab treatment of UC has achieved an outstanding breakthrough therapeutic effect.

According to relevant data published by ASCO in 2021, a total of 17 of the 19 enrolled patients completed at least one efficacy evaluation, and 16 of them achieved remission, with an overall ORR of 94.1% (Sheng et al., 2021).

## 5. Safety and tolerability

The safety and tolerability of RC48 in cancer patients have been evaluated in clinical trials, and the results showed that approximately 94.7% of patients started to experience adverse events (AEs) in the first 2 days after treatment, and most commonly mild AEs mainly included gastrointestinal diseases, fever, fatigue, and hematologic toxicity, while the most common grade 3 or worse side effects comprising neutropenia, leukopenia, hypesthesia, and increased conjugated blood bilirubin. Moreover, the incidence of ≥ grade 3, higher side effects, and death was resemble to that of the resemble drug T-DM1 (Zhu et al., 2021; Sheng et al., 2021). What’s more, the most serious AEs and dose-limiting toxicity-related AEs were observed only in the high-dose group (2.5 mg/kg and 3.0 mg/kg) cohort, suggesting that the AEs of RC48 were dose-dependent (Xu et al., 2021). More importantly, interstitial lung disease (namely is pneumonitis) was previously described as a rare but common AEs in some anti-HER2 drugs, involving trastuzumab(9.9%), lapatinib (0.2%), T-DM1 (0.5%), DS-8201 (9.0%), and trastuzumab duocarmazine (7.7%) (Hackshaw et al., 2020; Shitara et al., 2020). Nevertheless, no treatment-related lung damage was reported in the clinical trial of RC48 (Xu et al., 2021). From our perspective, the toxicity of ADCs seems to be related to the stability of the conjugate in the bloodstream and the off-target effects of the payload.

## 6. The advantages of RC-48 compared with TDM-1 and DS-8201

In recent years, derived from the cooperation effects of HER2 suppression and chemotherapy, a novel class of drugs has progressed and obtained new advances. Therefore, we summarize the structure characteristic (Table 1) and the status and efficacy of several ADCs targeting HER2 entering clinical trials (Table 2). In addition, RC-48 has certain advantages over other approved ADCs. Firstly, the structure design of RC-48 has the advantage of improving the efficacy and ensuring safety, such as in terms of molecular construction, DS-8201 uses topoisomerase, which has a quick effect but large side effects, while RC-48 has low side effects and better safety. Antibody: RC-48 adopted a novel HER2 mab optimized for screening, which has a prefer appetency to HER2 targets than trastuzumab and that has the potential to treat cancers with low or even unstable HER2 expression. Linker: Mc-VC-pa was invented for priority steady in human plasma and high-performance cleavage by human cathepsin, the enzyme lysate ligand is used to improve the overall killing effect of tumor tissue based on the "by-kill effect", and the lysate is easily controlled to improve the blood stability and safety of ADC drugs [Li et al., 2016]. Coupling way: Random coupling of cysteine is used, which is more homogeneous than lysine. Cytotoxin: Cytotoxin was also changed from dentin analog to MMAE, MMAE is a derivative of auristatin, which blocks cell cycle arrest through the aggregation of tissue microtubules bound to microtubules. Secondly, compared with the HER2 antibodies of other HER2-ADC drugs, RC-48 has better endocytosis and which is independent of V-ATPase activity and has no lysosomal resistance. Last but not least, RC48-ADC is extremely cytotoxic at very low concentrations, which was its major advantage; and the better medicinal properties of RC48-ADC and reduction in off-target toxicity.

**Table 1.** Comparison of RC-48 with T-DM1 and DS-8201 in structure.

ADC		Kadcyla (T-DM1)	Enhertu (DS-8201)	Disitamab vedotin (RC-48)
Antibody		trastuzumab	Trastuzumab	Hertuzumab
Linker	Name	SMCC	BC-GGFG-OH	MC-VC-PAB
	Conjugate method	Heterogeneous binding	Site-specific conjugate	Site-specific conjugate
	Cleavable	No	Yes	Yes
	DAR	3.5:1	8:1	4:1
payload	Released payload	mertansine (DM-1)	Deruxtecan (Dxd)	Monomethyl auristatin E (MMAE)
	Target	microtubule inhibitor	Topoisomerase I inhibitor	microtubule inhibitor
bystander killing effect		No	Yes	Yes

ADC: antibody-drug conjugates; DAR: drug/antibody ratio; SMCC: a non-cleavable small maleimidomethyl cyclohexane-1-carboxylate; BC-GGFG-OH: tert-butoxycarbonyl -glycyl glycyl phenylalanyl Glycine; MC-VC-PAB is a cathepsin cleavable ADC linker.

**Table 2.** Comparison of RC48 with T-DM1 and DS-8201 in clinical trials.

ADC	Tumor	Phase	ORR (%)	DCR (%)	m-PFS (months)	m-OS (months)	Reference	≥ grade 3 adverse events	Approved
T-DM1	BC	I	59.5	93.7	22.1	NR	D'Amico et al., 2019	Thrombocytopenia hepatic transaminitis anemia interstitial lung disease or pneumonitis	T-DM1 has been approved for the treatment of previously treated HER2-positive metastatic breast cancer.
	BC	II	25.9	–	4.6	–	Burris et al., 2011		
	BC	III	–	–	9.6	30.9	Verma et al., 2012		
	GC	III	–	–	3.2	5.8	Amiri-Kordestani et al., 2014		
	GC/GEJC	II/III	20.6	–	2.7	7.9	Thuss-Patience et al., 2017		
DS-8201	BC	I	59.5	93.7	22.1	NR	Tamura et al., 2019	decreased neutrophil count anemia decreased white-cell count interstitial lung disease or pneumonitis	The FDA granted accelerated approval to DS-8201a for patients with unresectable or metastatic HER2-positive BC who have received ≥2 prior anti-HER2-based regimens in the metastatic setting. DS-8201a has been approved for the treatment of adult patients with locally advanced or metastatic HER2-positive GC/GEJC who have received a prior trastuzumab-based therapy regimen.
	BC	II	60.9	97.3	16.4	NR	Modi et al., 2020b		
	GC/GEJC	I	43.2	79.5	5.6	7.0	Shitara et al., 2019		
	GC/GEJC	II	51	85.7	5.6	12.5	Hackshaw et al., 2020		
HER2-low							Hackshaw et al., 2020		
IHC2+/FISH − GC		I	26.3	89.5	4.4	7.8			
IHC1+ GC		I	9.5	71.4	2.8	8.5	Hackshaw et al., 2020		
HER2-low BC		I	37		11.1	29.4	Nguyen et al., 2021		
RC-48	Solid tumor	I	21	49.1	IHC2+/FISH- 35.7 IHC2+/FISH+ 20 IHC3 + 13.6	–	Xu et al., 2021	neutropenia leukopenia hypesthesia	In June 2021, RC-48 received its first Biologics License Application approval in China for the treatment of patients with HER2-overexpressing (defined as IHC2+ or 3+) locally advanced or metastatic GC (including gastroesophageal junction adenocarcinoma) who have received at least two systemic chemotherapy regimens.
	GC	I	21.3	46.8	–	–	Xu et al., 2021		
	GC	II	24.4	–	4.1	7.9	Peng et al., 2021		
	BC	Ib	1.5 mg/kg = 26.7 2.0 mg/kg = 46.7	96.7	–	–	Yaghoubi et al., 2021		
	BC	I	31.4	–	5.8	–	Cullinane et al., 2020		
	UC	I	50	100	–	–	Doi et al., 2017		
	UC	II	51.2	–	6.9	13.9	Yorozu et al., 2020		
	UC	II	46.9	–	4.3	14.8	Sheng et al., 2021		
HER2-negative		II	25	75	–	–	Sharma et al., 2017		
IHC1+ /IHC0 UC									
RC48+ toripalimab		UC	I/II	80	90		Sharma et al., 2020		

ADC: antibody-drug conjugates; T-DM1: trastuzumab emtansine; DS-8201: trastuzumab deruxtecan; RC48: disitamab vedotin; ORR: objective response rate; DCR: disease control rate; m-PFS: median progression-free survival; m-OS: median overall survival; NR: not reach; BC: HER2 + breast cancers; GC: HER2 + gastric carcinoma; UC: HER2 + urothelial cancer; GEJC: HER2 + gastric and gastroesophageal junction carcinoma.

## 7. Ongoing clinical trials

As a novel agent, RC48 as monotherapy or adjuvant treatment in clinical practice for the therapy of other cancer in the world, including UC in China and the USA, biliary tract cancer (BTC), non-small cell lung cancer (NSCLC), and HER2+ and HER2-low expressing BC in China (Table 3). More importantly, RemeGen announces US FDA and China have granted breakthrough therapy designation for RC48 in UC, and a novel drug application for UC was approved.

**Table 3.** Current RC48 clinical trials for multiple autoimmune disease.

Register number	Disease	State of the cancer	HER2 state	Therapeutic schedule	Clinical phase	Region
CTR20202569	GC	A	IHC 2+/3+	monotherapy	III	China
CTR20180844	GC	A	IHC 2+/3+	monotherapy	II	USA
CTR20200646	BC	A	IHC 1+	monotherapy	III	China
CTR20180492	BC	A with liver metastasis	+	monotherapy	III	China
CRT20161035	BC	A	IHC 1+ or +	monotherapy	Ib	China
CTR20182469	UC	A	IHC 2+/3+	monotherapy	II	China
ChiCTR2200055403	UC	A	not detected	combination therapy	II	China
CTR20192667	UC	A	+	monotherapy	II	USA
CTR20190939	NSCLC	A	(IHC2+/3+) or mutation	monotherapy	Ib	China
CTR20192057	BTC	A With failure of first-line chemotherapy	(IHC 2+/3+)	monotherapy	II	China
CTR20212908	MM	A	IHC 2+/3+	monotherapy	IIa	China
CTR20211602	GMT	A	+ OR IHC 1+	monotherapy	II	China

BC: breast cancer; GC: gastric cancer; UC: urothelial cancer; BTC: biliary tract cancer; NSCLC: non-small cell lung cancer; MM: malignant melanoma; GMT: gynecologic malignant tumor.

A: the state of the cancer is locally advanced or metastatic; IHC 2+/3+: HER2 overexpression; IHC 1+: HER2 low expression; +: HER2 positive; -: HER2 negative.

## 8. Future perspective and challenges

At present, ADC is an excellent and speedily growing field of targeted therapy for cancer, which combines the ability of monoclonal antibodies to specifically target oncology with a strong effect on killing activity. Although RC48 presented capacity against several HER2+ solid tumors, which have certain AEs and there are still things to be optimized. Firstly, to make more HER2-positive tumor patients benefit from RC48 drugs, the following aspects are the future research directions: further clarifying the anti-cancer mechanism and the mechanism of organ damage, enhancing targeting, and eliminating toxic side effects from off-target effects. Secondly, despite the ever, so the perspective anti-cancer effect of ADCs armed with microtubule-destabilizing warheads, there remains a clinical challenge in further enhancing ADC efficacy and conquering resistance mechanisms, worse internalization and/or invalid trafficking of the ADC compound and low tumor-associated surface antigens expression along with the expression of drug efflux pumps and/or multi-drug resistance transporters (D'Amico et al., 2019). Thirdly, specific PK and PD research should be executed on RC48 for the bystander effect and other profiles, while identifying predictive biomarkers and providing mechanistic insights to support clinical decision making. Fourthly, the prognosis of HER2-positive tumors has improved with the treatment of ADC drugs; however, cancer cells develop drug resistance and patients eventually deteriorate. There are usually two mechanisms of resistance: primary resistance and acquired resistance, the former is often caused by mutations in target genes, while the latter are usually classified into HER2-dependent resistance and HER2-independent resistance. Further studies should be conducted to clarify the possible mechanisms of resistance to RC48 in HER2-positive tumors, and take corresponding countermeasures to solve drug resistance, so that more tumor patients benefit.

HER2 plays essential roles in the pathogenesis of multifarious oncology, such as BC, GC, UC, and NSCLC, making them distinguished candidate targets for novel therapies. RC48 is a HER2-directed ADC, burgeoning as an effective tactic for tumor therapy, which not only heightens antitumor immunity in prior animal models but also boosts clinical effect for patients such as with GC, UC, and HER2 low-expressing BC. In June 2021, China authorized Biologics License Application (BLA) on the RC-48 for the therapy of HER2+ (IHC2+/3+) locally advanced or metastatic GC/GEJC who have prior accepted at least two systemic chemotherapy agents (Deeks, 2021). In January 2022, it is gratifying that BLA announced the approval of RC48 in patients with HER2+(IHC2+/3+) locally advanced or metastatic UC who have previously accepted chemotherapy in China. As a result, in the near further, likely RC48 will also have the potential to be approved by FDA and National Medical Products Administration heighten to treat other carcinomas, such as BC, GC, and UC as well NSCLC in the United States and China.
